# Adopt This Measure in the Smoky City

**Published:** 1896-07

**Authors:** 


### ADOPT THIS MEASURE IN THE SMOKY CITY.

AN ideal ordinance[1] for the regulation and control of the milk traffic of Pittsburg has been introduced in the Councils of that city, and its many excellent features should win for it the support of every milk-consumer of the entire city.

At first reading one might be led to think that it imposes unreasonable restrictions upon the producers; but this is only apparent, not real, and has its force only from the fact that we have so long been tolerant in allowing this raw food product to come to us unquestioned and unchallenged; while we have been straining our efforts to avoid, by cooking and other measures, certain dangers possessed by our drinking water and foods that bear only an insignificant comparison to milk as a dangerous food product in itself when used as a raw production, and which by its remarkable susceptibility to invasion by germs and other impurities fills the rôle of a vehicle of transmission, aside from the fact that we have remained in blissful ignorance of the physical condition of the animals producing our milk-supply, as well as the absolute lack in many instances of a single sanitary safeguard. The wealth of general information now before our people on this all-important topic makes it nothing short of criminal to permit the existing conditions to hold on a day longer than the time required to put into execution measures to control and remove these far-reaching dangers. Every veterinarian in Pennsylvania should give this his unselfish and unqualified support.

Let the good work go on.

